# Improving food system outcomes with beans, peas and pulses: a prospective mapping review of research in the UK

**DOI:** 10.1017/S1368980025101791

**Published:** 2026-01-12

**Authors:** Laura Lane, Rebecca Wells, Christina Vogel, Christian Reynolds

**Affiliations:** 1 Centre for Food Policy, City St George’s, University of London, London, UK; 2 MRC Lifecourse Epidemiology Centre, University of Southampton, Southampton, UK

**Keywords:** Food systems, Sustainable diets, Mapping review, Legumes, Pulses

## Abstract

**Objective:**

To map the spread of research on legumes, including beans, peas and other pulses; to identify research gaps and opportunities relating to the use of legumes for improved human nutrition, health and environmental outcomes; and to develop a novel method for clarifying research priorities.

**Design:**

Prospective mapping review, identifying and mapping ongoing research (2019–2023) across the value chain.

**Setting:**

UK.

**Participants/sample:**

Academic research studies in three databases.

**Results:**

Fifty ongoing research projects were identified, revealing a focus on the two ends of the value chain: production (twenty-one projects) and consumption (twenty-one projects). Only four projects encompassed the entire value chain from producer to consumer. Research on production includes the role of legumes in crop rotations for soil health and reduced fertiliser use, productivity interventions and improved breeds. Research on consumption includes dietary and health outcomes, predominantly cardiometabolic impacts, and legumes as an alternative protein source. Few projects focused on the middle of the value chain (four projects on product development) with none focused on processing, food service or retail.

**Conclusions:**

Further interdisciplinary projects, linking producers to consumers and with a greater focus on middle-chain actors, are needed. The food processing/manufacturing, food service and retail sectors hold significant power in food systems practice and governance. They play a crucial role in transitioning to a healthier and more sustainable food system. Understanding the drivers and barriers for these food systems actors in increasing production and consumption of beans, peas and pulses is required to inform future food policy and practice.

There is more evidence than ever before that our food system is connected to major contemporary challenges, from increasing rates of food insecurity, nutrient deficiency, obesity and non-communicable diseases to climate change, soil degradation, water pollution and biodiversity loss^(1–3)^. To address the rising burden of diet-related health issues, manage the significant environmental impacts of food production and tackle food insecurity for a growing global population, we must shift towards healthier and more sustainable dietary patterns^(4–6)^. A change in diets, however, is just part of the solution^(7)^. The availability and accessibility of foods that support healthy and sustainable diets are influenced by a complex network of actors and activities within our food system^(8–10)^. The need for food systems transformation to achieve better outcomes for both people and the planet is now undeniable^(11)^. This transformation will require actors across the food system to adapt their activities and the reassessment of policies to send clear signals to food system actors^(12)^.

Beans, peas and other pulses such as chickpeas and lentils, which are all types of legumes, have been identified as an accessible and affordable solution to our global health and environmental challenges^(13,14)^. There are at least fifty types of edible legumes consumed around the world. There is a broader range of legumes that are not grown for human consumption, including non-food legumes such as clover or vetch, that can be included in agricultural systems as animal feed^(15)^ or for their wider benefits for the environment or agricultural production systems^(16)^.

Legumes for human consumption have been identified as the food group associated with the greatest increase in life expectancy^(17)^. Their high dietary fibre content, in particular, is linked to a reduced risk of mortality and lower incidence of a range of non-communicable diseases^(18)^. There is a growing body of evidence for the nutrition and health benefits of legumes, mostly relating to beans and pulses, which are high in protein and fibre, low in saturated fat and sodium and rich in a broad range of micronutrients, particularly iron, zinc and folate^(19,20)^. A systematic review found that daily intakes of 150 g of pulses (minimum-maximum: 54–360 grams per day (g/d); cooked) are associated with increased satiety, healthier body composition, improvements in blood pressure, blood lipid profile and inflammation biomarkers and a lower risk of cardiovascular disease (CVD)^(21)^. Wider health benefits include improved gut microbiota composition and activity and reduced risk of type 2 diabetes and several types of cancer^(22)^. Recommended intakes vary across international food-based dietary guidelines^(23)^. Some refer to legumes, and some to pulses, resulting in a confused body of terminology across dietary guidelines^(24)^. Generally, 100 g of cooked pulses per d has been suggested to promote nutritional adequacy across a range of micronutrients^(25)^, and one cup of pulses daily makes a substantial contribution to daily fibre intakes^(26)^. At 21 g/d, the global mean daily consumption of all types of legumes indicates they are under-consumed in many nations^(27)^. UK consumption data is scarce, but two UK government datasets show that intakes are suboptimal. Mean daily purchases of beans, peas and other pulses are 28 g/d^(28)^, while mean daily consumption of beans and pulses (excluding peas) is 13 g/d per person^(29)^.

Legumes form a key component of sustainable diets, and their production supports a wide range of improved environmental outcomes^(4,26,30)^. While many types of legumes are grown for animal feed^(15)^, they can provide an alternative to meat and other animal sources of protein in human diets. Legume production typically has much lower environmental impacts, and notably lower greenhouse gas emissions, than animal-sourced protein^(31,32)^. This is primarily because legumes form a symbiotic relationship with soil bacteria called rhizobia. Rhizobia allow legumes to ‘fix’ nitrogen from the atmosphere into the soil, reducing the need for fertiliser and improving soil quality and productivity^(33)^. As fertilisers contribute significantly to agricultural greenhouse gas emissions, growing legumes can help to reduce agricultural contributions to climate change^(34)^. Supporting reductions in fertiliser can also improve water quality by reducing the impacts of run-off from farmland soils into rivers and streams^(35)^. Additionally, there is evidence that legume cropping systems can reduce farmland biodiversity loss^(36,37)^. The wider environmental benefits of growing legumes, known as ‘ecosystem services’, hold great promise to increase the environmental sustainability of European food systems^(16)^. Despite these significant benefits, production of edible legumes in Europe is low, with heavy reliance on imports to meet consumer demand^(15,38)^.

Evidence for the benefits associated with increased legume consumption and production has sparked a significant increase in research interest and activity across multiple disciplines^(36,39,40)^. But the extent of current research on legumes has not been documented. This lack of clarity prevents effective planning of future research and increases the possibility of duplication of effort. Therefore, the aim of this review was to understand the scope and spread of academic research on legumes underway or registered in the UK and to identify research gaps or opportunities. This was achieved using a novel prospective mapping review methodology, to clarify future research priorities in food systems and other research fields. The focus of this mapping review was edible legumes – namely beans, peas and other pulses. Given the known complexities in balancing the production of legumes for human consumption with the demands for animal feed and ecosystem services, a food systems approach was adopted, and the wider category of legumes was included.

## Methods

### Study design: Prospective mapping review of research in the UK

The design of this study was a prospective mapping review. This approach is an adaptation of traditional mapping reviews, which retrospectively map and categorise existing published literature on a topic^(41)^. A prospective mapping review is a novel methodology that spans multiple disciplines and anticipates the scope of forthcoming evidence on a topic, acting as a route to identifying research gaps. In this study, projects commenced or in progress in the last five years were identified and mapped against the value chain to visualise research gaps across the food system. This method supports other ‘horizon scanning’ or foresight research methods applied in food and health research^(42,43)^.

**Figure 1.**
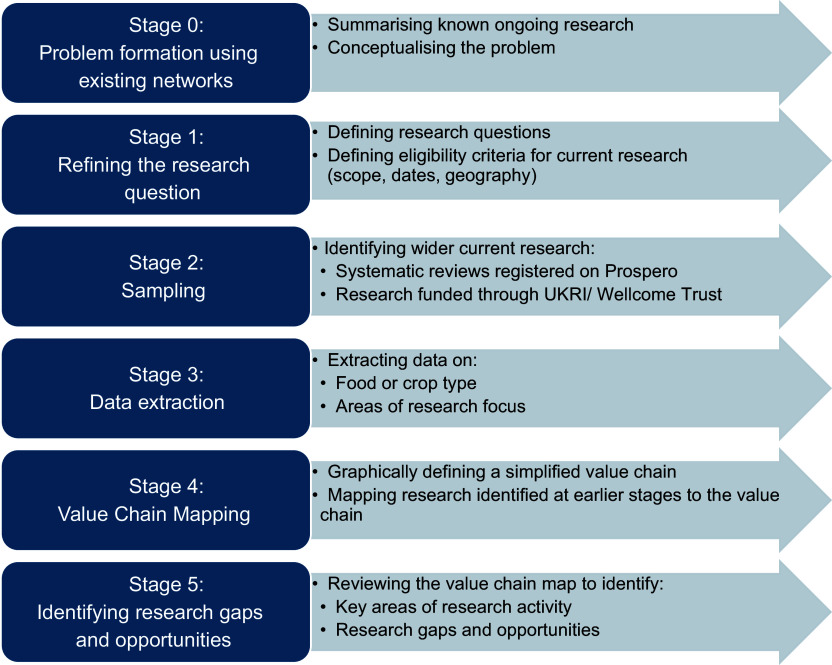
Prospective mapping review stages.

An adapted version of the mapping review method was applied to address the research aims of this study and followed a six-stage process, summarised in Figure 1. All stages were conducted by one researcher. Ethical approval was not required for this study because no human participants were involved.

### Stage 0: Problem formation through existing networks

Stage 0 was the problem formation stage for this prospective mapping review. An event, the ‘Beans as a Vehicle for Food System Transformation’ summit in September 2023 formed the basis of this stage. The event was not arranged by the research team but was co-convened by the BeanMeals research project^(40)^, Beans is How^(14)^ and the Agrifood for Net Zero Network^(44)^. This event brought together researchers, projects, companies and other interested parties working to raise the profile of beans; case studies were presented and research priorities across the food system were explored^(45)^. In November 2023, a second event, the ‘Options for Change’ workshop, was convened by BeanMeals, bringing together similar stakeholders to progress discussions within the emerging community of interest. These events provided insights into research activity in the UK, highlighting specific research teams, where the research was occurring (both geographically and along the value chain), and the types of food being researched. At this stage, it was not clear to the authors or the wider community of interest what other research was underway in other academic settings, and it was difficult to identify research gaps. Further stages were subsequently designed to map the research underway or planned.

### Stage 1: Refining the research questions

Using the eligibility criteria in Table 1, the research questions for this review were defined as: (1) What academic research on beans, peas, pulses and other legumes is underway or registered in the UK?

**Table 1.** Eligibility criteria

	Inclusion criteria	Exclusion criteria	Rationale
Scope	All legumes	Peanuts	All legumes are included given the inconsistent use of terminology. Oil-rich peanuts are excluded from the list of non-edible legumes as they have a very different nutrition profile to other types of legumes^(24)^.
Research type	Academic research	Commercial or third sector (NGO) research	The method is based on mapping reviews, which typically focus on published academic literature^(41)^.
Dates	Started 2019–2023		Defined to capture new research projects or unpublished work in progress.
Geography	Research conducted in, or in collaboration with, UK academic institutions Systematic reviews registered in the UK using PROSPERO	Research not conducted in, or in collaboration with, UK academic institutions Systematic reviews registered in the UK using PROSPERO	All research identified through UKRI/Wellcome was included, even where the research itself was not conducted within the UK. Likewise, all international systematic reviews registered in the UK-based PROSPERO register were included, irrespective of the geographical location of the research as the outcomes (e.g. health outcomes) are relevant to the UK food system.

(2) What is the scope and spread of this research across the value chain, and where are the research gaps or opportunities?

### Stage 2: Sampling

During November 2023, a desktop review of research activities was completed using three UK research portals. The search terms used are summarised in Table 2. The portals accessed were PROSPERO – International prospective register of systematic reviews (https://www.crd.york.ac.uk/PROSPERO/), UKRI Gateway to publicly funded research and innovation (https://gtr.ukri.org/) and Wellcome Trust Funding (https://wellcome.org/grant-funding/people-and-projects/grants-awarded). Eligibility criteria are set out in Table 1. All searches were limited to English language projects underway between 2019 and 2023. All research identified through UK Research and Innovation (UKRI)/Wellcome was included, even where the research itself was not conducted within the UK. Likewise, all studies registered in the UK-based PROSPERO register were included, irrespective of the geographical location of the research.

**Table 2.** Portals and search terms

Research portal	Search terms	Rationale	Notes
PROSPERO (Prospective registry of systematic reviews in health and social care)	legume* OR bean* OR Pea OR Peas OR Pulses	Title only to assess reviews focused on these foods Notes on search terms: ‘Pulse’: Returns mostly cardiovascular studies; ‘Pea’ or ‘Peas’ returns more appropriate content than ‘Pea*’	30 reviews were identified, 20 of which were identified as relevant or potentially relevant. Reviews were excluded where search terms were used in different (non-food) contexts (e.g. pulse rate in humans).
UKRI gateway to publicly funded research and innovation	Separate searches: Legume, Fabaceae, Lentil*, Pulse, Pea, and Bean*, limited to ‘Active’ projects	As defined for PROSPERO	769 projects were identified, duplicates removed and screened for relevance. 26 projects were identified as relevant or potentially relevant.
Wellcome Trust Funding	Separate searches: Pulse or pulses; Legume or legumes; Bean or beans; Pea or peas; fabaceae	A search of all grants awarded from current and past grant holders (4288 records)	0 projects were identified as relevant or potentially relevant.

### Stage 3: Data extraction

For each research project identified as meeting the inclusion criteria, data on food/crop type and value chain stage were extracted. The area of research focus was also defined thematically by the authors. Data were extracted in Microsoft Excel and represented visually using Datawrapper^(46,47)^.

### Stage 4: Value chain mapping

A highly simplified visual representation of the UK value chain for beans, peas and pulses was developed following Stage 0. Development of this visual map was informed by wider literature on value chain mapping^(48)^. Few edible legume varieties are grown in the UK, and only fava (faba) beans and peas are grown commercially at scale^(49)^. The supply chains for other types of beans, peas and pulses, which meet the majority of UK demand through import, can be long and fragmented^(50)^. The purpose of the visual representation therefore involved mapping broad categories of activities that take place in the UK from production or import to consumption and waste disposal. A detailed and complex value chain map for a wider range of legume crops was beyond the scope of this study. Research identified at Stages 0–3 was mapped to the value chain, using the area of research focus identified from project abstracts at Stage 4. If research projects included more than one activity in the value chain, all activities were included.

### Stage 5: Identifying research gaps and opportunities

The main clusters of research activity, potential research gaps and opportunities were identified using the value chain diagram and described narratively.

## Results

**Figure 2.**
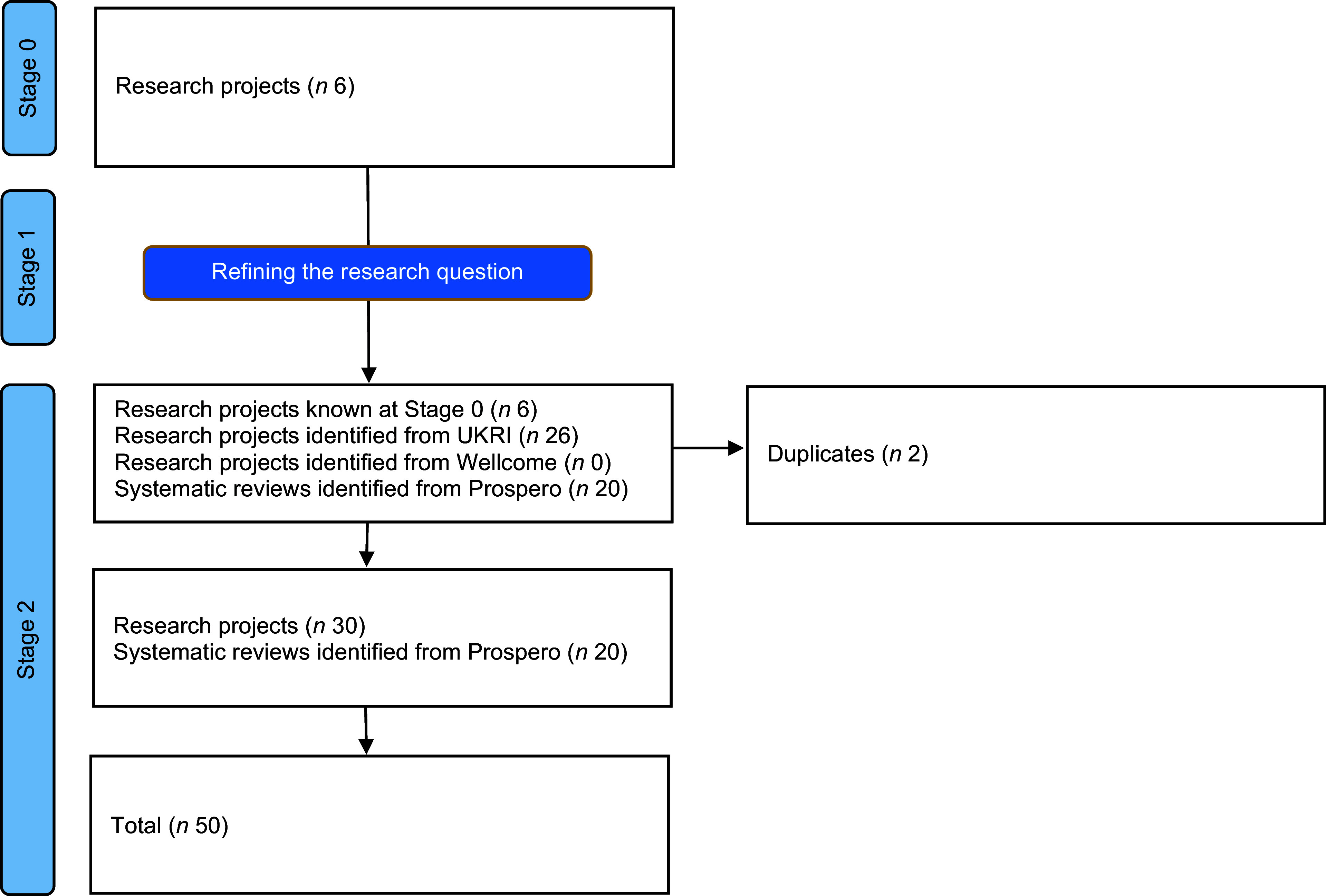
Summary of UK research activities ongoing or starting in 2023, broken down by the stages of this prospective mapping review.

A total of fifty research projects met the inclusion criteria, comprising twenty systematic reviews and thirty research projects. Figure 2 provides a flow chart of the research activities, adapted from PRISMA^(51)^.

The six research projects identified at Stage 0 are summarised in Table 3: BeanMeals^(40)^ and Raising the Pulse^(39)^, both of which form part of the Transforming UK Food Systems Programme^(52)^; research at the Warwick Crop Centre^(53)^ on new varieties of haricot beans; research on legumes at the James Hutton Institute^(54)^; The British On-Farm Innovation Network’s work on nitrogen-efficient plants for Climate Smart arable cropping systems^(55)^; and research at the Quadram Institute on novel ingredients from chickpeas^(56)^.

**Table 3.** UK academic research activity on beans, peas and pulses known at stage 0

Research	Lead institution(s)	Summary	Food/crop	Areas of research focus
BeanMeals^(40)^	Universities of Oxford, Warwick, Hertfordshire, Hull and Liverpool	‘Fork-to-farm’ approach to food system transformation Mainstreaming new varieties of UK-grown beans into healthy school and home-cooked meals Developing and analysing systemic innovations, requiring collaboration between multiple actors, for reducing the consumption of products high in fat, sugar and salt using UK-grown beans Codesigned with Leicestershire-based local partners including schools, businesses and the Local Enterprise Partnership https://www.eci.ox.ac.uk/beanmeals	UK-grown haricot beans (Godiva and Capulet)	UK Food System (UKRI-funded ‘Transforming UK Food Systems Programme’)
Raising the Pulse^(39)^	University of Reading	Systems analysis of the environmental, nutritional and health benefits of pulse-enhanced foods Enhancing white bread with UK-grown faba bean flour as a replacement for soya as a flour improver Encouraging food manufacturers to use UK pulses to satisfy consumer demand for plant-based and pulse-rich foods, rather than importing chiefly soya-based ingredients https://ukfoodsystems.ukri.org/research-projects-training-reports/raising-the-pulse-rtp/	UK-grown faba bean	UK Food System (UKRI-funded ‘Transforming UK Food Systems SPF Programme’)
Warwick Crop Centre Research on beans^(53)^	University of Warwick	Working to develop new varieties of haricot beans since 2011 ‘Capulet’ and ‘Godiva’ are the first newly registered varieties of bean from this research Currently being trialled with a commercial seed company on UK farms, as well as in the BeanMeals project https://warwick.ac.uk/fac/sci/lifesci/wcc/research/food/beans/	UK-grown haricot bean	Agricultural production (new varieties)
Nitrogen-efficient plants for Climate Smart arable cropping systems (NCS)^(55)^	The British On-Farm Innovation Network (BOFIN)	Farmer-led research, including 17 industry and research partners and over 200 farmers Enabling UK farming to bring about a reduction of 1·5 Mt CO_2_e per annum (54 % of the maximum potential for the industry) Aiming to increase legume cropping in arable rotations from 5 to 20 % across the UK and replace up to 50 % of imported soya meal in livestock feed with homegrown legumes Delivering significant benefits for crop and livestock productivity, including cost savings of over £1bn/year https://bofin.org.uk/the-ncs-project/	Legumes	Agricultural production (environmental outcomes)
Multiple research projects, for example, RADIANT^(54)^	James Hutton Institute	Legume-supported crop systems with a focus on underutilised crops and novel cropping systems Innovating beyond the farm gate to encourage resource use efficiency, improved ecosystem functions and realisation of circular economies https://www.hutton.ac.uk/department/ecological-sciences/agroecology/	Legumes	Agricultural production (environmental outcomes)
PulseON Foods^(56)^	The Quadram Institute, King’s College London	Deriving a novel ingredient from chickpeas Using specially developed milling and drying processes to preserve the cellular structure and make starch more resistant to digestion, with benefits for blood glucose control https://www.pulseonfoods.com/	Chickpeas	Product development (dietary and health outcomes)

### Research Question 1: Identifying the scope and spread of academic research on legumes underway or registered in the UK

The desktop review undertaken at Stage 2 identified twenty systematic reviews relating to legumes through PROSPERO. An additional twenty-six relevant research projects funded by UKRI were identified. No relevant projects were funded by the Wellcome Trust. After deduplication, a total of fifty research activities met the defined inclusion criteria, comprising twenty systematic reviews and thirty research projects, as summarised in Figure 2.

**Figure 3.**
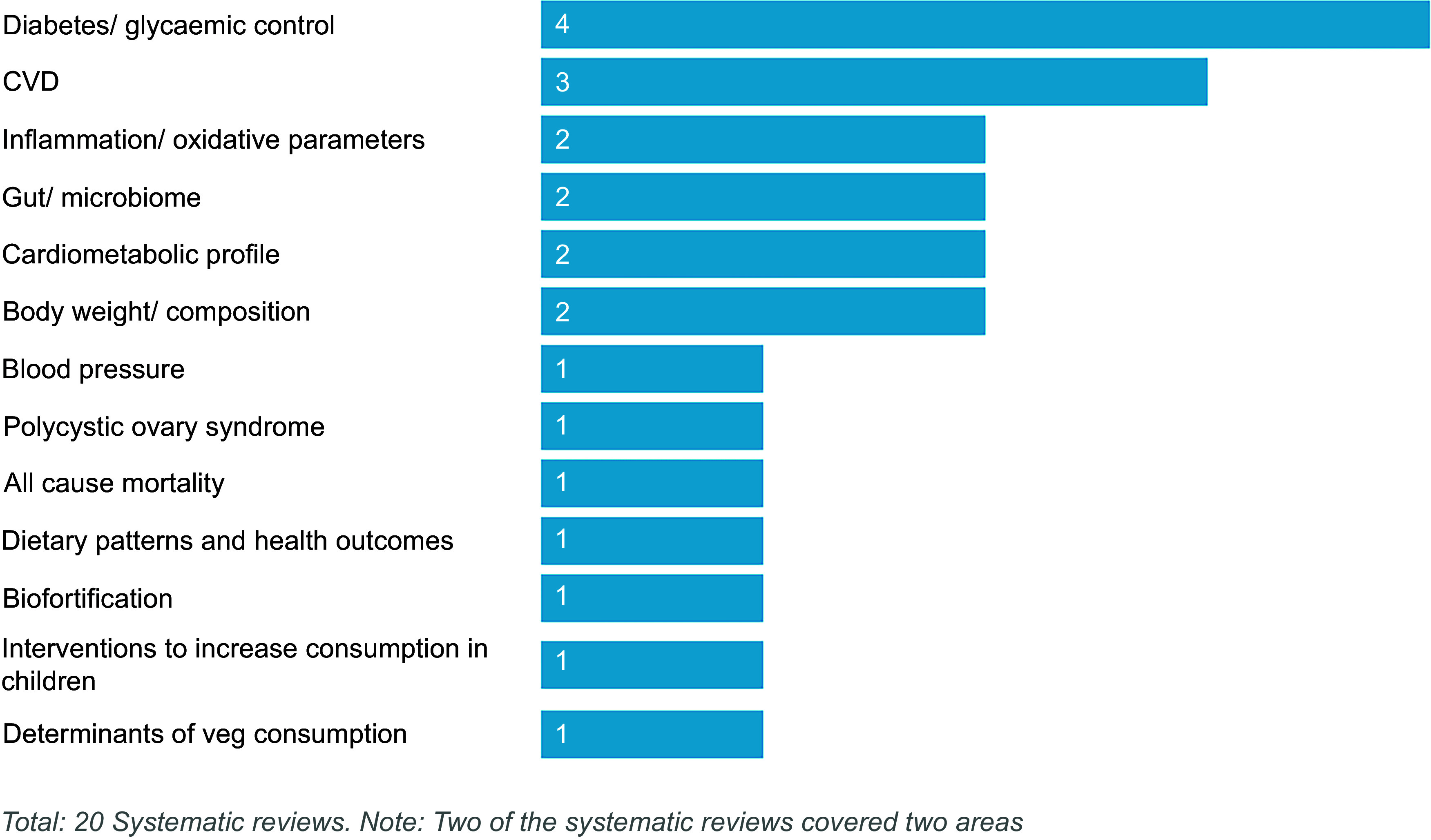
Systematic reviews (2019–2023) relating to legumes, by theme.

The details of each of the fifty included research studies are presented in Table 4. Of the twenty systematic reviews included, most focused on health outcomes associated with overall intake of ‘legumes’ (*n* 12) rather than specific types of beans (*n* 4), pulses (*n* 4) or peas (*n* 0). Key areas of research interest included diabetes/glycaemic control (*n* 4), CVD (*n* 3), inflammation and oxidative parameters (*n* 2), gut health/microbiome (*n* 2) and body weight/body composition (*n* 2). Other areas of interest being examined by single systematic reviews included biofortification, blood pressure, cardiometabolic profile, polycystic ovary syndrome, all-cause mortality, approaches to increase consumption among children and determinants of vegetable consumption, including beans (Figure 3). Only one of the identified systematic reviews was being conducted in the UK. Other systematic reviews were being conducted by researchers in China (*n* 3), Iran (*n* 3), Australia (*n* 3), Canada (*n* 3), Greece (*n* 2), New Zealand (*n* 1), Brazil (*n* 1), Portugal (*n* 1) and multi-country (non-UK) teams (*n* 2). The scope of these reviews extends beyond legumes grown and consumed in the UK, but nutrition and health impacts associated with consumption are universally applicable. They are included here because they are registered in the UK-based PROSPERO register; irrespective of study location, this analysis of nutrition/health outcomes contributes to the wider international evidence base that impacts UK food policy and practice.

**Table 4.** Summary of systematic reviews (2019–2023) relating to legumes identified through PROSPERO

Authors	Title	Year	Country	Food or Crop	Areas of research focus
Chiavaroli, Grant, Ayoub-Charette *et al*.	Effect of different types of dietary pulses on established therapeutic lipid targets for cardiovascular risk reduction: an updated systematic review and dose–response meta-analysis of randomised controlled trials http://www.crd.york.ac.uk/PROSPERO/display_record.asp?ID=CRD42023432826	2023	Canada	Pulses	CVD
Kolomvotsou & Tsitsou	‘Effects of legumes and legume-based products consumption on cardiometabolic profile : a systematic review’ http://www.crd.york.ac.uk/PROSPERO/display_record.asp?ID=CRD42023462542	2023	Greece	Legumes	Cardiometabolic profile
Reyneke, Beck, & Lambert	Examining the Prevalence of Legumes within Dietary Patterns Associated with Anti-Inflammatory Effects: An Umbrella Review http://www.crd.york.ac.uk/PROSPERO/display_record.asp?ID=CRD42023406661	2023	Australia	Legumes	Inflammation/oxidative parameters
Seyyedin & Mohammadi-Sartang	The effects of soyabean products on metabolic factors of PCOS women http://www.crd.york.ac.uk/PROSPERO/display_record.asp?ID=CRD42023427930	2023	Iran	Soya	Polycystic ovary syndrome
Shaygantabar & Alirezaei	Effect of non-soya legumes consumption on inflammatory and oxidative parameters http://www.crd.york.ac.uk/PROSPERO/display_record.asp?ID=CRD42023451736	2023	Iran	Non-soya legumes	Inflammation/oxidative parameters
Silva &Abreu	Effectiveness of nutrition interventions to increase pulses consumption among children and adolescents: a systematic review and meta-analysis http://www.crd.york.ac.uk/PROSPERO/display_record.asp?ID=CRD42023417668	2023	Portugal	Pulses	Interventions to increase consumption in children
Reynolds, Hossain, Agyei *et al*.	Legume intake on cardiometabolic profile and gut microbiome function in adults with and without diabetes: a systematic review and meta-analysis of randomised controlled trials http://www.crd.york.ac.uk/PROSPERO/display_record.asp?ID=CRD42023456953	2023	New Zealand	Legume	Cardiometabolic profile and gut/microbiome
Seetha, Boy, Mehta *et al*.	Can iron biofortified common beans consumption reduce iron deficiency anaemia and improve physical work performance? – a systematic review and meta-analysis of randomised controlled trials http://www.crd.york.ac.uk/PROSPERO/display_record.asp?ID=CRD42022323108	2022	Colombia, India, USA	Beans	Biofortification/iron deficiency
Wang, Zhao, Miao *et al*.	Effects of White Kidney Bean Extract (Phaseolus vulgaris L.) on weight and body composition: a systematic review and meta-analysis of controlled clinical trials http://www.crd.york.ac.uk/PROSPERO/display_record.asp?ID=CRD42022355744	2022	China	Kidney Bean	Body weight/composition
Zargarzadeh, Mousavi, & Esmaillzadeh	Legume consumption and risk of all-cause and cause-specific mortality: Protocol for a systematic review and dose-response meta-analysis of prospective studies http://www.crd.york.ac.uk/PROSPERO/display_record.asp?ID=CRD42022296260	2022	Iran	Legumes	All-cause mortality
Mendes, Niforou, Ververis *et al*.	Intake of legumes and CVD: a systematic review and dose-response meta-analysis http://www.crd.york.ac.uk/PROSPERO/display_record.asp?ID=CRD42021247565	2021	Greece	Legume	CVD
Milhomens, Domene, De Lucca Da Silva *et al*.	Determinants of the consumption of fruits, vegetables, rice and beans in low- and middle-income countries: a rapid review http://www.crd.york.ac.uk/PROSPERO/display_record.asp?ID=CRD42021275446	2021	Brazil	Beans	Determinants of veg consumption
Reyneke, Beck, Neale *et al*.	Effect of consumption of non-oilseed legumes on blood pressure in adults: a systematic review of randomised controlled trials as evidence for promoting legumes in Australian Dietary Guidelines http://www.crd.york.ac.uk/PROSPERO/display_record.asp?ID=CRD42021237732	2021	Australia	Non-oilseed legumes	Blood pressure
Thorisdottir, Arnesen, Bärebring *et al*.	Consumption of pulses/legumes and risk of CVD and type 2 diabetes and their risk factors: a systematic review http://www.crd.york.ac.uk/PROSPERO/display_record.asp?ID=CRD42021250084	2021	Finland, Iceland, Norway, Sweden	Pulses/legumes	CVD and diabetes
Bielefeld, Grafenauer, & Rangan	The Effects of Legume Consumption on Markers of Glycaemic Control: A Systematic Literature Review of Randomised Controlled Trials http://www.crd.york.ac.uk/PROSPERO/display_record.asp?ID=CRD42020179734	2020	Australia	Legume	Glycaemic control
Harding, Marinangeli, Zafron *et al*.	A systematic review of the effect of dietary pulses on microbial populations inhabiting the human gut http://www.crd.york.ac.uk/PROSPERO/display_record.asp?ID=CRD42020167811	2020	Canada	Pulses	Gut/microbiome
Keats, Charbonneau, & Oh	Frequency, variety, and quantity of specific complementary foods (fruits and vegetables, animal-source foods, and pulses, nuts and seeds) and their impact on dietary and health outcomes among children 6–23 months of age: a systematic review and meta-analysis http://www.crd.york.ac.uk/PROSPERO/display_record.asp?ID=CRD42020203655	2020	Canada	Pulses	Dietary patterns and health outcomes
Hafiz, Bösch, Orfila *et al*.	The impact of legume consumption on indices of glycaemic control – a systematic review and meta-analysis http://www.crd.york.ac.uk/PROSPERO/display_record.asp?ID=CRD42019162322	2019	UK	Legume	Glycaemic control
Jiang, Zhang, Liu *et al*.	Relationship between legumes consumption and metabolic syndrome: a meta-analysis of observational studies http://www.crd.york.ac.uk/PROSPERO/display_record.asp?ID=CRD42019131777	2019	China	Legume	Metabolic syndrome
Tang, Wan, & Zheng	Associations between legumes and soya intake and incident type 2 diabetes: a meta-analysis of prospective studies http://www.crd.york.ac.uk/PROSPERO/display_record.asp?ID=CRD42019126403	2019	China	Legume, soya	Diabetes

*Note*: Projects identified at https://www.crd.york.ac.uk/PROSPERO/ in November 2023.

**Figure 4.**
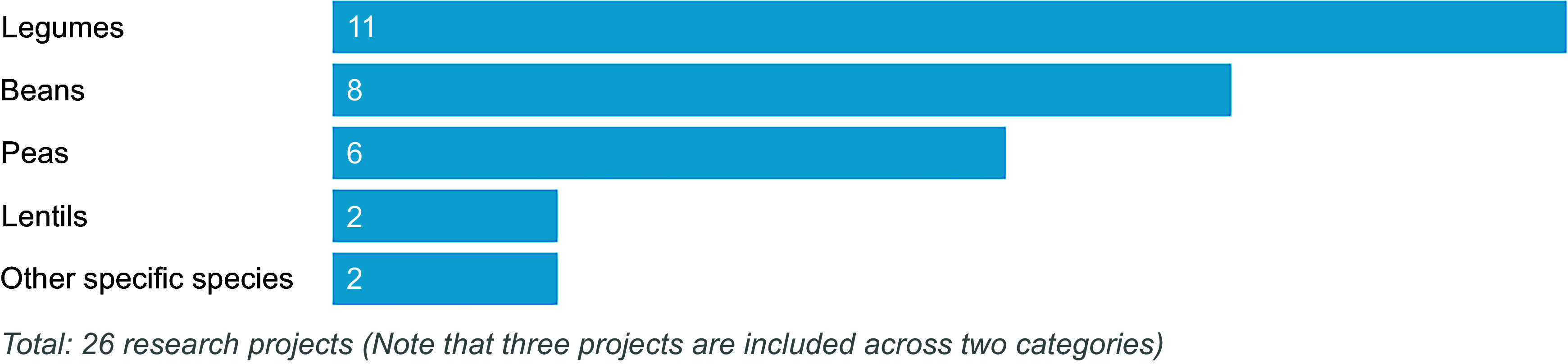
UKRI-funded research (2019–2023) related to legumes, by crop.

**Figure 5.**
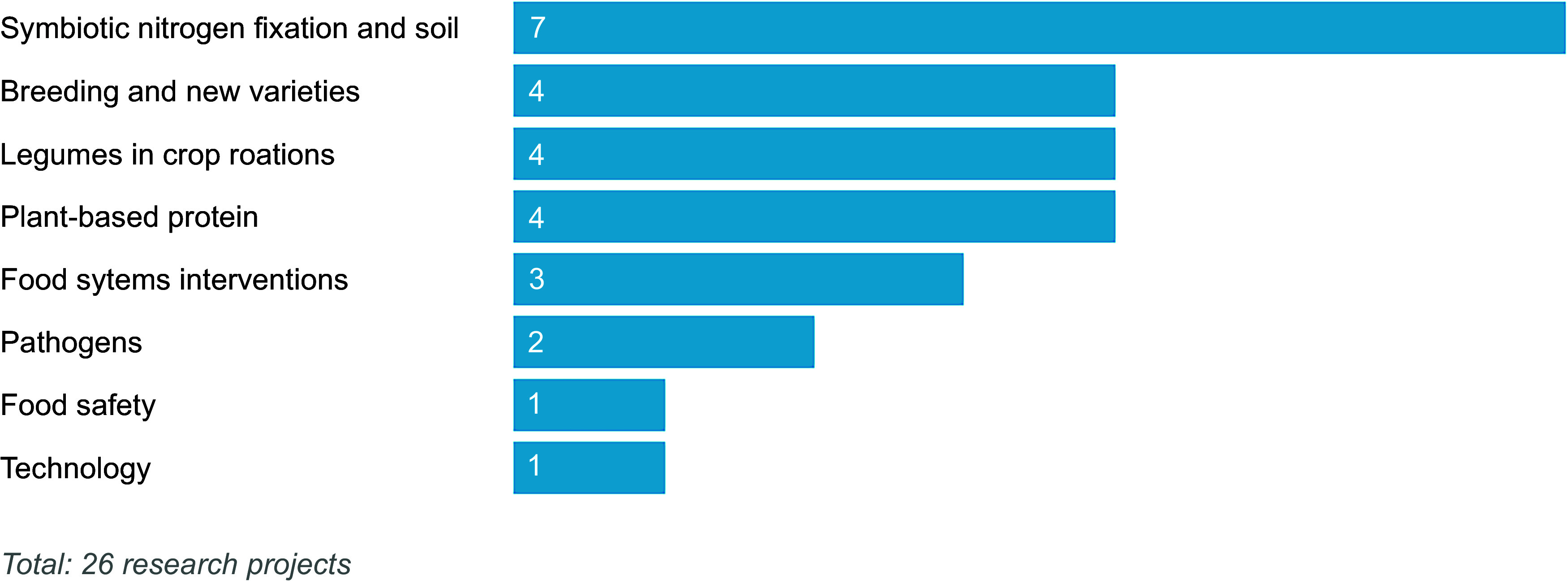
UKRI-funded research (2019–2023) relating to legumes, by area of research focus.

The twenty-six UKRI-funded research projects identified that related to legumes included research grants (*n* 8), EU-funded projects (*n* 6), studentships (*n* 11) and collaborative R&D projects (*n* 1). The research projects are detailed in Table 5 and presented by crop in Figure 4. Most studies focused on the general category of legumes (*n* 11) rather than beans (*n* 8), peas (*n* 6), lentils (*n* 2), other species such as lupins or clover (*n* 2) or pulses (*n* 0). When grouped by area of research focus (Figure 5), most projects (*n* 7) focused on interventions to increase crop productivity, including the use of legumes in crop rotations to reduce fertiliser inputs and improve soil health through symbiotic relationships with soil bacteria (rhizobia). Breeding and new varieties (*n* 4) and options for plant-based proteins (*n* 4) were also considered. Few (*n* 4) projects take a food systems perspective. For example, food systems approaches are not explicitly mentioned, or projects do not encompass the entire value chain from producer to consumer. Wider systems actors, activities or outcomes are also generally not considered in planned project analyses or syntheses. Two projects adopting a food systems approach were funded through UKRI’s ‘Transforming UK Food Systems Strategic Priorities Fund (SPF)’^(52)^, namely BeanMeals^(40)^ and Raising the Pulse^(39)^. The other two projects consider broader international food systems. One of these projects was the ZIRON Pulse project, which considered upscaling the adoption and exploitation of iron and zinc rich beans by rural populations in Africa. The other project, HOLiFOOD, integrated food safety risk analysis across the food system, with European lentils as a case example.

**Table 5.** Summary of UKRI-funded projects relating to legumes (2019–2023)

Project types	Funder	Lead Institution	Title	Summary	Food/crop	Areas of research focus
Collaborative R&D	Innovate UK	Processors and Growers Research Organisation (PGRO) and University of Nottingham	Development and provision of a digital platform enabling industry to predict harvest date and yield of vining pea	Maximising yield through predictive technologies https://gtr.ukri.org/projects?ref=10033310	Peas	Production (technology)
Research grants	Strategic Priorities Fund (SPF)	University of Reading	‘Raising the Pulse’: Systems analysis of the environmental, nutritional and health benefits of pulse-enhanced foods	Enhancing white bread with UK-grown faba bean flour as a replacement for soya as a flour improver https://gtr.ukri.org/projects?ref=BB%2FW017946%2F1	Faba bean	Food system (product development)
		University of Oxford	‘Thinking beyond the can’: Mainstreaming UK-grown beans in healthy meals (BeanMeals)	Developing and analysing systemic innovations, requiring collaboration between multiple actors to mainstream two new varieties of UK-grown beans into healthy school/home-cooked meals https://gtr.ukri.org/projects?ref=BB%2FW017733%2F1	Bean	Food system (production/product development)
	Global Challenges Research Fund (GCRF)	James Hutton Institute	ZIRON Pulse	Upscaling adoption and exploitation of a wide diversity of Fe- and Zn-rich beans by rural populations in Africa https://gtr.ukri.org/projects?ref=BB%2FT008865%2F1	Beans	Food system (international production)
	Biotechnology and Biological Sciences Research Council (BBSRC)	University of Sheffield	Peptidoglycan remodelling during the Rhizobium leguminosarum life cycle, from the rhizosphere to the formation of bacteroids	Rhizobial symbiosis in peas and beans for reduction in fertiliser use (sustainable agriculture) https://gtr.ukri.org/projects?ref=BB%2FW013800%2F1	Peas and beans	Production (symbiotic nitrogen fixation)
		National Institute of Agricultural Botany (NIAB)	EAGLE: Enhanced Analytical and Genetics Tools for Improving UK Food Legumes	Developing new resilient, nutritious UK-grown legume varieties https://gtr.ukri.org/projects?ref=BB%2FW01923X%2F1	Legumes	Production (breeding/new varieties)
			Bean Enhance	Tracking pathogens of the common bean in Uganda and Tanzania https://gtr.ukri.org/projects?ref=BB%2FT012692%2F1	Common bean	Production (pathogens)
	Horizon	University of Reading	LegumeLegacy – Optimising multiple benefits of grass, legume and herb mixtures in crop rotations: modelling mechanisms and legacy effects	Legumes in ruminant-based production systems: yield, quality and environmental impacts https://gtr.ukri.org/projects?ref=EP%2FX028003%2F1	Legumes	Production (production systems)
	Economic and Social Research Council (ESRC)	University of Glasgow	Using consumption and reward simulations to create desire for plant-based foods	Cognitive psychology and labelling (with beans and lentils given as examples of plant-based foods) https://gtr.ukri.org/projects?ref=ES%2FT011343%2F1	Beans and lentils	Consumption (plant-based protein)
EU-funded	Horizon Europe Guarantee	PGRO	Breeding European Legumes for Increased Sustainability (BELIS)	Increasing the competitiveness of the legume breeding industry and designing efficient delivery of the genetic progress to key stakeholders (7 food crops and 7 animal forage crops) https://gtr.ukri.org/projects?ref=10072967	Legumes	Production (breeding)
		Legume Generation consortium, John Innes Centre/University of Aberystwyth	Boosting innovation in breeding for the next generation of legume crops for Europe	Innovation across 6 legume species https://gtr.ukri.org/projects?ref=10073595	Soyabean, lupins, pea, lentil, common bean, clover	Production (breeding)
		Scotland’s Rural College (SRUC)	Developing intercropping for agrifood value chains and ecosystem services delivery in Europe and Southern countries	27 participants from 15 countries (3 continents), with one of the themes covering legume/cereal intercropping. Focus on soil health and greenhouse gas mitigation https://gtr.ukri.org/projects?ref=10061194	Legumes	Production
		University of Reading	LEGUMINOSE: Legume-cereal intercropping for sustainable agriculture across Europe	Understanding barriers to legume-based intercropping and increasing acceptance for farmers. Farm-based trials (7 European countries including UK, Egypt, and Pakistan) https://gtr.ukri.org/projects?ref=10039837	Legumes	Production (crop rotations)
		Newcastle University/Queen’s University of Belfast	HOLiFOOD: Holistic approach for tackling food system risks in a changing global environment	Integrated food safety risk analysis (RA) framework in Europe, looking across the food system, with lentils as one of three case study foods https://gtr.ukri.org/projects?ref=10040684 https://gtr.ukri.org/projects?ref=10041445	Lentil	Food system (food safety)
		James Hutton Institute/University of Dundee	Root2Res: Root phenotyping and genetic improvement for rotational crops resilient to environmental change	Enhanced resilience to and mitigation of climate change, with the inclusion of legumes and lentils as an emerging crop https://gtr.ukri.org/projects?ref=10041427 https://gtr.ukri.org/projects?ref=10039664	Lentils	Production (breeding)
Studentships	Natural Environment Research Council (NERC)	University of Sheffield	Bacterial cell envelope remodelling in Rhizobium leguminosarum: contribution to symbiosis and resistance to abiotic stress	Symbiosis in pea and bean, with a focus on biochemistry https://gtr.ukri.org/projects?ref=studentship-2744522	Pea and bean	Production (symbiotic nitrogen fixation)
	BBSRC	University of Edinburgh	The role of legumes in achieving net zero: reducing nitrogen losses and improving soil health	Focus on soil health and reduction of fertiliser. Nitrogen-fixing legumes (non-food crops) for ecosystem services https://gtr.ukri.org/projects?ref=studentship-2754265	Red clover, black medic, lucerne	Production (symbiotic nitrogen fixation)
		Brunel University London	Designer plant burgers – use of targeted biochemistry and chemistry to generate flavour (taste and aroma) during extrusion of plant protein	Pea protein isolate in plant burgers (engineering and flavour science) https://gtr.ukri.org/projects?ref=studentship-2743988	Pea	Product development (plant-based protein)
		University of Oxford	The regulation of the legume symbiosis with nitrogen-fixing bacteria	Better understanding of legume symbiosis https://gtr.ukri.org/projects?ref=studentship-2600903	Legumes	Production (symbiotic nitrogen fixation)
		University of Bristol	Understanding protein valuation – the key to reducing meat consumption?	Three groups of protein sources were compared: meat, meat-alternative products and naturally occurring plant-based sources (e.g. legumes) https://gtr.ukri.org/projects?ref=studentship-2573007	Legumes	Consumption (plant-based protein)
		University of East Anglia	Root legume symbioses signalling	Regulation of root legume symbioses https://gtr.ukri.org/projects?ref=studentship-2444186	Legumes	Production (symbiotic nitrogen fixation)
			Investigations of the link between the bacterial cytoskeleton and N-cycling in the Rhizobium-legume symbiosis	Microbial molecular biology technologies. Rhizobium-legume symbiosis https://gtr.ukri.org/projects?ref=studentship-2246559	Legumes	Production (symbiotic nitrogen fixation)
			Data science to feed a changing world: superior drought tolerance in nutritious traditional beans	Hyperspectral imaging and genomics methods to screen the response to water deficit stress of common bean landraces from the Andes https://gtr.ukri.org/projects?ref=studentship-2578607	Common bean	Production (breeding)
		University of Oxford	Control of gene expression in rhizosphere bacteria by plant metabolites	Expanding the legume/bacterial symbiotic relationship to other species: biological nitrogen fixation in cereal crops https://gtr.ukri.org/projects?ref=studentship-2270043	Legumes	Production (symbiotic nitrogen fixation)
	Engineering and Physical Sciences Research Council (EPSRC)	University of Nottingham	Food microstructure, sustainable protein	Microstructure and functionalities of sustainable protein, with legumes as one option for plant-based protein https://gtr.ukri.org/projects?ref=studentship-2277655	Legumes	Product development (plant-based protein)
	ESRC	University of East Anglia	Identifying and exploiting the novel sources of genetic resistance for root diseases of pea	Genetic resistance to the root rot complex, the most destructive disease of pea, endemic across most pea-growing areas of the UK https://gtr.ukri.org/projects?ref=studentship-2589571	Peas	Production (pathogens)

### Research Question 2: Clarifying research gaps and opportunities

**Figure 6.**
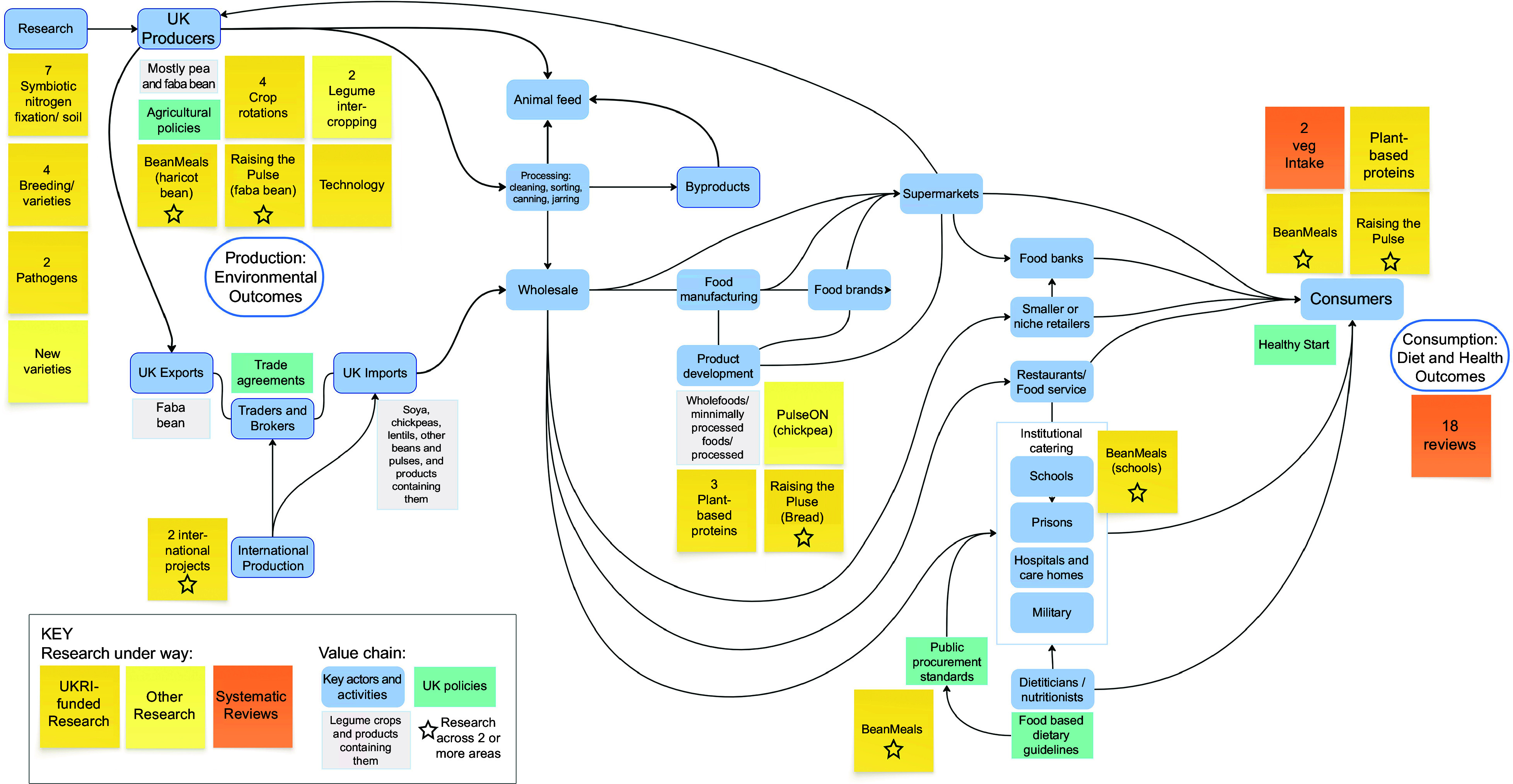
Research activities on legumes (2019–2023) mapped to the UK value chain and food system outcomes.

Mapping of the included research activities related to beans, peas, pulses and other legumes identified across the value chain is presented in Figure 6. The results show a striking pattern with the included research studies largely populating the two ends of the value chain: production (*n* 21) and consumption (*n* 21). Environmental outcomes are typically investigated at the production stage and dietary/health outcomes at the consumption stage. Production-focused research is linked to improved breeds (i.e. improved nutrition composition or drought tolerance/resilience to climate change); interventions to increase productivity (i.e. linked to the symbiotic relationship with bacteria for nitrogen fixation in soil); and the role of legumes in crop rotations, with a focus on reduced fertiliser and soil health. Consumption-focused research is focused on improving dietary and health outcomes of increased legume consumption, with a focus on cardiometabolic health impacts including diabetes/glycaemic control and CVD. At the middle of the value chain, a smaller number of research studies (*n* 4) are exploring new product development. These studies included developing novel plant-based foods including using pulses as an alternative protein source to meat from animals, a novel ingredient derived from chickpeas and white bread made with the addition of UK-grown faba bean flour.

Existing research activities focused on beans, peas, pulses and other legumes, which are registered or being conducted in the UK, rarely take a ‘food system’ perspective or encompass the entire value chain from producer to consumer. Only four of the fifty included studies adopted this approach. None of the identified studies had a primary focus on the role of middle-chain actors such as food processors, manufacturers or wholesalers in increasing legume use across our food systems. The clear research gaps are illustrated in Figure 6. The role of food providers (e.g. food service, institutional catering and retail sectors) is limited in existing research activities. The role of public sector caterers has been included in one study, BeanMeals, which trialled bean-based school meals in Leicestershire with beans from local supply chains^(40)^. This project is the only one identified that explicitly focused on UK policy. Other potential research gaps, as illustrated in Figure 6, include the role of imports to the UK, or the impact of the diversion of leguminous crops to animal feed rather than food for humans.

## Discussion

This prospective mapping review showed that research on legumes (beans, peas, pulses and a broader group of legumes not typically grown for human consumption) being conducted or registered in the UK targets the two ends of the value chain: production and consumption. The existing evidence base indicates the potential for legumes to improve environmental and human health outcomes by increasing the production and consumption of these foods, respectively. This existing evidence base may, at least in part, explain the current research focus on these areas of the value chain. A smaller number of studies consider new product development in the centre of the value chain. These studies used pulse-based ingredients in novel food products. This research area is likely to expand in future years, given consumer interest in plant-based foods^(57)^.

In seeking to identify gaps and opportunities for future research activities, these results highlight that current research efforts on legumes in the UK rarely encompass the entire value chain from producer to consumer or adopt a food systems perspective. No existing academic research is primarily focused on the role of middle-chain actors such as processors, manufacturers, wholesalers, food brands and retailers, and research on food providers (e.g. institutional catering) is limited. The involvement of these middle-chain actors – linking the value chain from producer to consumer – can support the transition to improved food system outcomes^(58)^. Supermarkets and the hospitality industries, in particular, have the power to influence the food supply chain, at both farm production and consumer levels^(59)^. The big market players have significant weight in these networks of relationships, exerting influence across food systems and the policy decisions affecting them^(58,60)^. A lack of connected action on agriculture and nutrition, termed the ‘missing middle’, has been identified as a barrier to the achievement of the Sustainable Development Goals^(61)^.

Research on imports to the UK, or the impacts of promoting legumes for animal feed rather than for human consumption, is also limited. This gap is significant because, in the UK, most of the legumes we eat are imported, and most of what we grow is fed to animals. This situation impacts production and consumption in the UK and is consistent with patterns across Europe^(15,16)^.

Multiple barriers across our food systems can limit the production, processing, marketing and consumption of legumes^(33)^, and a lack of coordinated effort to increase production and consumption in parallel has been cited as a key barrier to change^(49)^. European crop systems, public policies and market dynamics have historically promoted cereals to the detriment of legumes^(34)^. In contrast to what is needed for human health, post-Brexit UK agricultural policy directs legume production towards nitrogen-fixing break crops or animal feed^(62)^. There is a significant lack of incentives to grow legumes for human consumption in the UK, despite government targets for achieving net zero and reducing diet-related diseases. This policy disconnect may be one of the biggest barriers to change. Arguably, the existing research focus on improving production and consumption of legumes is unlikely to yield the necessary food systems transformation to improve planetary and human health, because it does not fully consider existing policy and governance frameworks. Policy incentives and wider support for change, especially among middle-chain actors, are needed.

Wider barriers to increased production in the UK include (i) a lack of defined markets for novel legume crops, which poses a financial risk to growers, (ii) resistance from food manufacturers to rely on UK-based legume supply chains without a consistent, high-quality supply and (iii) limited processing capacity for UK-grown crops, such as cleaning, sorting and canning produce^(63)^. Systemic efforts are required to address and overcome these barriers to achieve the desired beneficial food systems outcomes associated with increased legume production and consumption.

Research that links together a range of actors and activities across the food system to collaboratively overcome existing challenges for increased production and consumption has been tested. The TRUE collaboration (TRansition paths to sUstainable legume-based systems in Europe), which links academic researchers, producers, agronomists, processors, associated businesses and decision-makers, is a good example of previous multidisciplinary efforts to increase sustainable legume cultivation^(64)^. In the UK, the BeanMeals project adopted a ‘fork-to-farm’ approach, including linking the whole value chain from children’s lunchtime meals in schools in Leicestershire to producers for new varieties of British beans. This highly integrated approach to research is rare, potentially because the short supply chains in this project are quite different from the typically long and fragmented value chains for beans. BeanMeals identified that eight steps are required to get a bean from North America, where it might typically be sourced, to a school meal in Leicestershire. This assessment therefore mapped the ‘missing middle’ of the value chain^(65)^ that was absent from most studies identified in the current prospective mapping review. A subsequent report from the project identified three potential pathways for legume promotion in the UK: (i) community enterprise, (ii) artisanal entrepreneurs and (iii) food giants. All three pathways include a broad range of actors, but the report outlined the need to enhance research and evidence on the role of the missing middle in order to boost legume production and consumption in the UK^(63)^.

Changemakers looking to drive population-level dietary shift for the benefit of people and the planet, including a greater intake of beans, peas and pulses, must therefore look beyond the dietary choices of consumers and consider the broader range of actors and activities involved in food access and availability. Collaborative, multi-sector efforts could bring significant benefits in linking producers to consumers, driving greater involvement by middle-chain actors. To support this approach, there is a role for collaborative capacity builders that link together networks with shared goals^(36,66)^. Leading the way is the international ‘Beans is How’ network. Their Theory of Change for doubling global bean consumption includes promoting the role of social media influencers, chefs in restaurants and the wider food service industry in shaping consumer demand for pulses^(14)^. Non-governmental or charitable organisations also have a valuable role. WWF (World Wide Fund for Nature), for example, has developed guidance for food retailers and the food service sector to support dietary shift towards ‘planet-based diets^(67,68)^. Nutritionists and public health professionals working to improve population diet can act as collaborative capacity builders by promoting the inclusion of these foods in the diet, thereby helping to deliver improved environmental outcomes as well as vital improvements to dietary health.

### Future research

Future research should prioritise studies that would enable detailed value chain mapping for specific types of beans, peas and pulses, including both imported and UK-grown crops. Detailed mapping would provide useful insights into the barriers and opportunities to boost legumes across the value chain. A wide range of stakeholders across the food system should be involved to co-create detailed maps of the value chains and, crucially, support the identification of appropriate policy interventions. The mapping and policy activities would benefit from including a wide range of legumes for animal consumption or environmental benefit, alongside beans, peas and pulses, to provide insights into the challenges associated with repurposing current production activities towards legumes for human consumption.

Future research on legumes should adopt a food systems approach, with interdisciplinary consideration of the whole value chain. Across the food system, there is a concentration of power in the input, processing and retail sectors^(69)^; further research could explore the role these ‘middle-chain’ actors could have on the production and consumption ends of supply chains. The food retail sector – and notably large supermarket chains – holds the potential to positively influence the food system for beans, peas and pulses, because the majority of food purchases are made in this sector^(70)^. Yet the specific role of this sector and associated potential policy interventions are underexplored. A scoping review of the role of the retail sector in supporting increased production and consumption of beans, peas and pulses is in progress^(71)^.

Companies in the middle of the value chain, such as product developers and manufacturers, food service companies and the retail sector, could play a pivotal role in progressing collaborative multi-sector efforts to expand the products that contain beans, peas and pulses. Company nutritionists, public health professionals and recipe developers can all play a starring role in promoting beans, peas and pulses in product development and promotion. The commercial realities, opportunities for change and examples of innovation should be evaluated, ideally by independent researchers. Such evaluations would provide examples of best practice and diffuse innovation while ensuring that conflicts of interest are minimised^(72–74)^.

### Study strengths and limitations

This research study proposes a novel method for identifying research gaps and opportunities. The prospective mapping review approach identifies and maps ongoing research to minimise duplication of emerging research areas. This new approach could be adopted by researchers working across a range of academic disciplines. It could also aid the development of collaborations with other groups starting similar research projects and help to clarify the focus of systematic literature reviews. This methodological approach is particularly relevant for new and emerging areas of research and evidence interest. The topic of ultra-processed foods may provide another example of where this approach could be applied. Mapping research across the value chain supports a wider focus on the network of actors and activities that are collectively responsible for delivering ‘farm to fork’ solutions. This approach aligns with broader systems thinking for food-related public health and environmental issues.

This study also has some methodological limitations. It provides only a snapshot of academic research activities in late 2023, and additional research projects will now be in development. PROSPERO provides information about registered systematic reviews and, though not limited to reviews focused on health and social care, research efforts across other disciplines (such as agriculture or economics) are less likely to be registered on this repository if the review is not obviously linked to human health outcomes. Scoping reviews and other forms of reviews are also not registered with PROSPERO. Future research could also include other platforms such as Dimensions. Ai (https://www.dimensions.ai/), Open Science Framework (OSF) (https://osf.io/) and Figshare (https://figshare.com/) to cover a greater breadth of ongoing research activity. UKRI and Wellcome research portals accessed in this study provide insights into funded research in the UK. Academic projects in receipt of grants from private sector entities were not included in this study because of the potential for conflicts of interest in the presentation of findings. Non-government-funded research being conducted outside of academia was beyond the scope of this study. Searches performed in this study therefore did not identify research being conducted at the Royal Botanic Gardens, Kew, for example, which is investigating legume crop resilience and adaptation to climate change. Activity within the private sector, such as the use of pulse-based ingredients in novel plant-based foods or commercial trials, or multi-sector collaborations through organisations such as Beans is How^(14)^ are alternative pathways to generate knowledge and evidence. Future prospective mapping reviews could therefore consider defining a broader set of inclusion criteria.

## Conclusion

Legumes, such as beans, peas and other pulses, hold significant potential to support improved food system outcomes. We need to grow and eat more legumes in the UK to achieve their potential health and environmental benefits. The results of this prospective mapping review are encouraging, showing a significant number of research studies underway that will provide evidence towards future food policy and practice change. Existing research activities, however, are largely isolated at either end of the value chain: production and consumption. Future research activities in this field should focus on (i) adopting food systems perspectives; (ii) examining the role of middle-chain actors that link producers to consumers; and (iii) reviewing the policy landscape that shapes the activities across the value chain.
